# Nanomaterial datasets to advance tomography in scanning transmission electron microscopy

**DOI:** 10.1038/sdata.2016.41

**Published:** 2016-06-07

**Authors:** Barnaby D.A. Levin, Elliot Padgett, Chien-Chun Chen, M.C. Scott, Rui Xu, Wolfgang Theis, Yi Jiang, Yongsoo Yang, Colin Ophus, Haitao Zhang, Don-Hyung Ha, Deli Wang, Yingchao Yu, Hector D. Abruña, Richard D. Robinson, Peter Ercius, Lena F. Kourkoutis, Jianwei Miao, David A. Muller, Robert Hovden

**Affiliations:** 1 School of Applied and Engineering Physics, Cornell University, Ithaca, New York 14853, USA; 2 Department of Physics & Astronomy, and California NanoSystems Institute, University of California, Los Angeles, California 90095, USA; 3 Department of Physics, National Sun Yat-Sen University, Kaohsiung 80424, Taiwan; 4 National Center for Electron Microscopy, Molecular Foundry, Lawrence Berkeley National Laboratory, Berkeley, California 94720, USA; 5 Nanoscale Physics Research Laboratory, School of Physics and Astronomy, University of Birmingham, Edgbaston, Birmingham B15 2TT, UK; 6 Department of Physics, Cornell University, Ithaca, New York 14853, USA; 7 Department of Materials Science and Engineering, Cornell University, Ithaca, New York 14853, USA; 8 Department of Chemistry and Chemical Biology, Cornell University, Ithaca, New York 14853, USA; 9 School of Chemistry and Chemical Engineering, Huazhong University of Science and Technology, Wuhan 430074, China; 10 Kavli Institute for Nanoscale Science, Cornell University, Ithaca, New York 14853, USA

**Keywords:** Nanoparticles, Fuel cells, Transmission electron microscopy

## Abstract

Electron tomography in materials science has flourished with the demand to characterize nanoscale materials in three dimensions (3D). Access to experimental data is vital for developing and validating reconstruction methods that improve resolution and reduce radiation dose requirements. This work presents five high-quality scanning transmission electron microscope (STEM) tomography datasets in order to address the critical need for open access data in this field. The datasets represent the current limits of experimental technique, are of high quality, and contain materials with structural complexity. Included are tomographic series of a hyperbranched Co_2_P nanocrystal, platinum nanoparticles on a carbon nanofibre imaged over the complete 180° tilt range, a platinum nanoparticle and a tungsten needle both imaged at atomic resolution by equal slope tomography, and a through-focal tilt series of PtCu nanoparticles. A volumetric reconstruction from every dataset is provided for comparison and development of post-processing and visualization techniques. Researchers interested in creating novel data processing and reconstruction algorithms will now have access to state of the art experimental test data.

## Background & Summary

Electron tomography attempts to reconstruct 3D objects from 2D projection images taken at different viewing angles, or tilts—producing the entire internal structure of a specimen or region of interest. Since the first 3D reconstruction from electron micrographs^1^, tomography with the scanning transmission electron microscope (STEM) has been widely applied to nanoscale materials^2–12^. Utilizing the sub-angstrom 2D resolution of modern STEM, 3D reconstructions with sub-nanometre and even atomic detail have been demonstrated^13–16^. In the design of advanced nanomaterials, 3D characterization of nano-scale structure offers valuable insight into a material’s macroscale function. As a result, demand for nanoscale STEM tomography is high^17^.

Routine tomographic methods face several challenges that reduce final quality. The geometry of most specimens and specimen holders restricts tilt range to less than roughly 140°. Commonly referred to as ‘the missing wedge’, an incomplete tilt-range limits the information available for reconstruction and manifests as an elongation in the final reconstruction^18,19^. Contamination and specimen radiation sensitivity limit the number of viewing angles and the signal to noise, which in turn, restricts the final resolution in 3D^20^. Depth-of-field limits the maximum allowable size of the object that can be reconstructed; a particular problem for aberration corrected STEM^13,21,22^.

Recently, efforts towards new reconstruction methods promise higher resolution reconstructions using fewer viewing angles and lower radiation doses than traditional reconstruction algorithms like Weighted Back Projection (WBP) and Simultaneous Iterative Reconstruction Technique (SIRT). New approaches such as the iterative Fourier-based equal slope tomography^23^ and compressed sensing inspired algorithms^24,25^ have demonstrated success in STEM tomography by improving reconstruction quality with reduced sampling. However, we still lack a fundamental understanding of when and how these algorithms fail. Adopting new algorithms into routine tomography requires thorough investigation^17^.

A lack of high-quality, open access data is impeding development and validation of new algorithms and software for 3D reconstruction, visualization, and analysis. Currently, the best tomographic datasets are harboured by a privileged few. Researchers best suited for creating novel data processing and analysis techniques do not readily have access to experimental data.

To address this deficiency, we present five datasets that have pushed the limits of electron tomography. Each dataset was acquired using a unique experimental technique, is of high quality, and contains materials with structural complexity:

Tom_1) Tomography of Hyperbranched Co_2_P Nanoparticle: a 150° tomographic tilt series, taken at 2° increments. The Co_2_P nanocrystal has a complex morphology of bundled branches that resembles a six-pointed star^26^. This dataset represents a tilt range and increment typical of nano-scale STEM tomography.

Tom_2) 180° Tomography of NPs on Nanofibre: a tilt series taken at 1° tilt increments over the full 180° tilt range of platinum nanoparticles on a graphitized carbon nanofibre support. This dataset provides a complete range of tilts, allowing researchers to better understand the effects of missing information. 16 fast acquisition images were acquired at each tilt, and the experimental signal-to-noise level can be adjusted by averaging different numbers of these images.

Tom_3) Atomic Resolution Tomography of Pt NP: a 145° equal slope tomography tilt series of a single platinum nanoparticle, acquired at atomic resolution, enabling reconstruction of atomic features^15^.

Tom_4) Atomic Resolution Tomography of Tungsten Needle: An equal slope tomography tilt series of the tip of a tungsten needle, acquired over the full 180° range, enabling atomic resolution reconstruction^16^.

Tom_5) Through-Focal Tomography of Pt-Cu Catalyst: a 138° through-focal tomographic tilt series acquired in an aberration-corrected microscope of Pt-Cu fuel cell catalyst nanoparticles with a complex internal pore structure on an extended carbon support at 3° increments^13^. This dataset overcomes the limited depth of field that accompanies high-resolution aberration corrected imaging^21^ by combining through-focal sectioning and tilt-series tomography to reconstruct extended objects.

The datasets include raw tilt series aligned for reconstruction and 3D reconstructions of each specimen—all in an easily readable TIF format.

Combined, these datasets provide a standard, open set of test data for the growing field of tomographic reconstruction and visualization. The datasets allow researchers to rigorously test their algorithms from alignment to reconstruction on real experimental data. The datasets will also find a use as a training tool for scientists new to tomography, a validation tool for 3D tomographic visualization, and a template to seed a future open library of tomographic data.

## Methods

In ADF-STEM electron tomography, a focused electron beam with sub-nanometre diameter is rastered across a sample of interest. Electrons scattered from the sample are recorded using an annular dark field detector, which generates a 2D projection image of the sample^27,28^.

The viewing angle is changed by rotating the specimen and a series of projection images from different angles is acquired. For the vast majority of electron microscopes, a single axis of rotation is permitted by the stage, although more complex tilt geometries have been demonstrated^17,29,30^. After each successive tilt during the experiment, the specimen moves relative to the electron beam and must be re-centred. The specimen can only be re-centred approximately at the time of acquisition and so the data is said to be ‘misaligned’. The end result of a tomographic experiment is a set of ‘misaligned’ STEM images corresponding to a specific specimen tilt.

Aligning the STEM images prior to reconstruction is vital to establish a common axis of rotation (i.e., tilt-axis) in the image series. Alignment methods include the use of fiducial markers^31,32^, cross correlation^33^, and centre of mass. Once aligned, a reconstruction algorithm is used to generate a 3D reconstruction of the sample.

**Figure 1:**
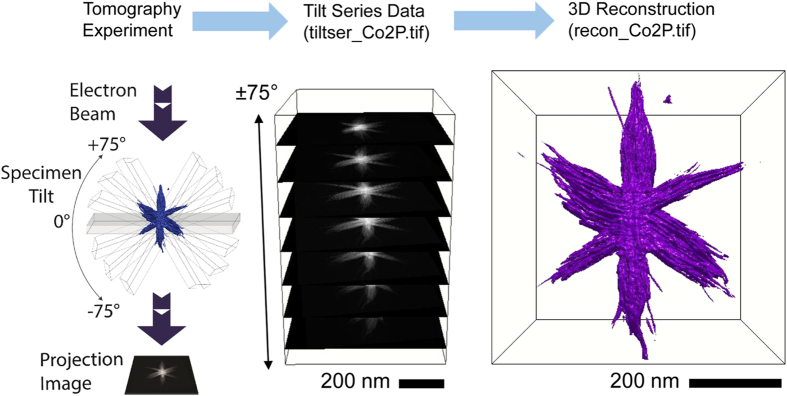
Illustration of electron tomography data acquisition and reconstruction process. Series of 2D images acquired of object of unknown 3D structure at different viewing angles. Images shown are from tiltser_Co2P.tif. 2D images combined into image stack ordered by viewing angle i.e., a tilt series. Tilt series is aligned, and reconstruction algorithm is applied to produce 3D reconstruction of object. A 3D isosurface visualization of recon_Co2P.tif is shown as an example rendered using tomviz.

The basic steps of the tomographic method are illustrated in Fig. 1.

### Tom_1: Tomography of hyperbranched Co_2_P nanoparticle

#### Sample preparation

Hyperbranched Co_2_P nanocrystal synthesis methods and scientific relevance are discussed in detail by Zhang *et al.*^26^ Samples were prepared for tomographic analysis by pipetting a drop of organic solution containing Co_2_P nanocrystals onto the surface of a copper TEM grid coated with an amorphous carbon film. Once the organic solution had dried, Co_2_P nanocrystals were dispersed over the grid. A drop of a solution of gold nanoparticles in water was then pipetted onto the grid, and allowed to dry. The gold nanoparticles were used as fiducial markers to align the tilt series.

#### Data acquisition

The tomographic tilt series of Co_2_P nanocrystals was acquired using an FEI Tecnai F20 scanning transmission electron microscope (STEM) at Cornell University. The microscope was operated at an accelerating voltage of 200 kV, with a convergence semi-angle of 9.6 mrad, and beam current of ~8–10 pA. This yields a nominal 2D resolution of up to 1.6 Å for STEM annular dark field (ADF) images. The tomographic tilt series was acquired over a 150° range at 2° intervals using a high angle annular dark-field (HAADF) detector. The scale in each image is ~0.71 nm per pixel.

#### Alignment and reconstruction

Each of the 76 projections in the tilt series, tiltser_Co2P.tif, has been aligned to a fiducial particle close to the Co_2_P nanocrystal using manual alignment techniques (except projections 50 and 51, which are blank to correct for 4° goniometer backlash during acquisition). Similarly, the tilt axis was determined by manually choosing the axis of rotation that minimized artifacts and maximized detail in the final reconstruction. We provide an example reconstruction, recon_Co2P.tif, produced from tiltser_Co2P.tif using the SIRT algorithm.

### Tom_2: 180° Tomography of nanoparticles on nanofibre

#### Sample preparation

Graphitized nanofibres, loaded with platinum nanoparticles at 10 wt. % were dispersed in a methanol solution and dried onto the tip of a tungsten omniprobe needle. The needle was inserted into a Fischione 2050 On-Axis Rotation Tomography Holder for data acquisition.

#### Data acquisition

One 93° tilt series and one 95° tilt series were acquired with an offset of ~85.3° between the viewing angle of the first image of the first series, and the viewing angle of the first image of the second series. The two tilt series together therefore cover slightly more than the full 180° tilt range. The overlapping region between the two tilt series was used to align them together in post-processing. The angular increment for each tilt series was 1°. In order to reduce scan noise and thus improve signal to noise ratio, 16 images, each with a 1 μs per pixel dwell time, were recorded at each viewing angle and saved as an image stack to be aligned in post processing. Data was acquired using an FEI Tecnai F20 scanning transmission electron microscope (STEM) at Cornell University. The microscope was operated in low angle annular dark field (LAADF) mode an accelerating voltage of 200 kV with a probe current of approximately 5 pA. A convergence angle of ~6.9 mrad was used to optimize resolution over a large depth of field. Images were acquired with 1024×1024 pixels. Non-orthogonality in probe scan direction was observed and was corrected in all images by applying a 0.6 degree shear parallel to the tilt axis with linear interpolation. The field of view in each image was 363.52 nm.

#### Alignment and reconstruction

**Figure 2:**
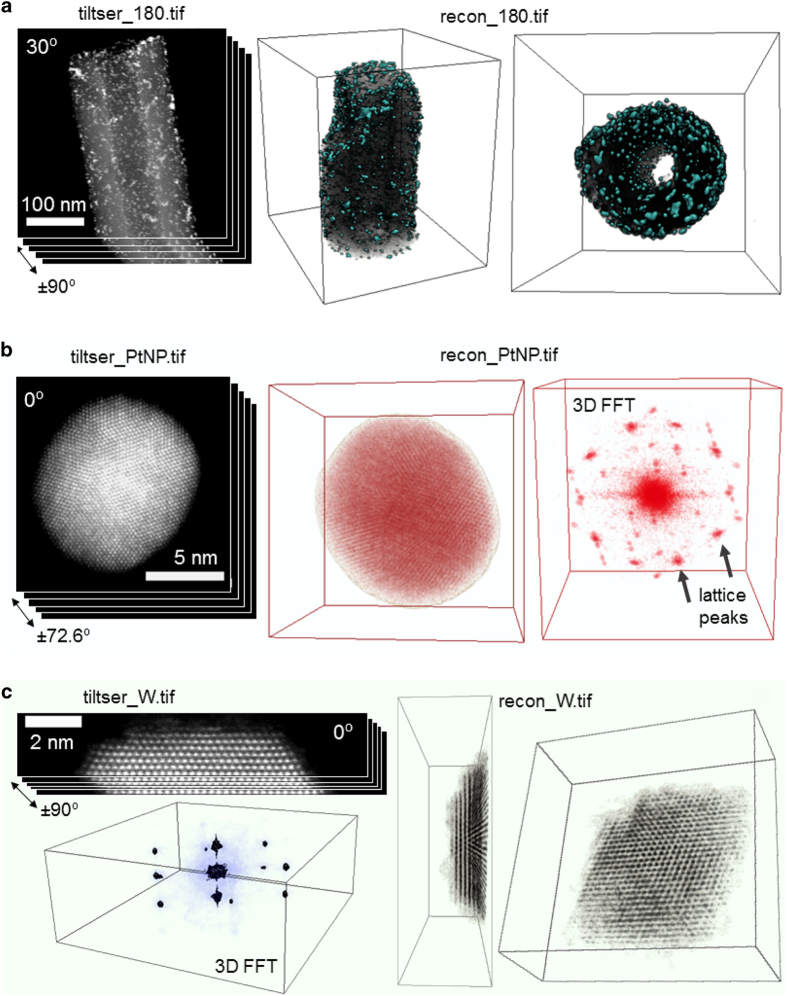
Illustrations of tilt series and sample reconstructions. (**a**) Sample image from tiltser_180.tif. Mixed 3D volume/isosurface visualizations of recon_180.tif show exterior of fibre, with nanoparticles visible on exterior, and hollow interior of nanofibre, containing nanoparticles. (**b**) Sample image from tiltser_PtNP.tif. Mixed 3D volume/isosurface visualization of recon_PtNP.tif and volume visualization of 3D Fourier transform of recon_PtNP.tif, showing platinum reciprocal lattice spots. (**c**) Sample image from tiltser_W.tif. Mixed 3D volume and isosurface visualization of recon_W.tif and of the 3D Fourier transform (cropped) of recon_W.tif, showing tungsten reciprocal lattice spots. All 3D visualizations produced using tomviz.

Each of the 1 μs per pixel image stacks of 16 images was aligned by cross correlation. Each stack was then summed to form a single image. The single images from both tilt series were then combined into a single 180° image stack, tiltser_180.tif, for reconstruction, with duplicate viewing angles discarded. The tilt series was aligned using a centre of mass method. A 3D reconstruction of the data, recon_180.tif, was produced using a weighted back projection algorithm. Data illustrated in Fig. 2a.

### Tom_3: Atomic resolution tomography of platinum nanoparticle

#### Sample preparation

Platinum nanoparticles were deposited onto a grid consisting of a 5-nm-thick silicon nitride membrane with dimensions of 100 μm×1500 μm, supported on a 100 μm-thick silicon frame designed for loading into a TEM (TEMwindows.com). High temperature coating of a 1–2 nm carbon layer was applied to mitigate charging effects due to the electron beam. The grid was then loaded onto a Fischione 2020 tomographic sample holder for data acquisition in the TEM.

#### Data acquisition

The tomographic tilt series of platinum nanoparticles was acquired using an uncorrected FEI Titan STEM at the University of California, Los Angeles. The microscope was operated with a beam energy of 200 keV, a 100 pA probe current, and a 10.7 mrad convergence semi-angle. A tilt series of 104 projections was acquired from a platinum nanoparticle with equal-slope increments and a tilt range of ±72.6°.

#### Alignment and reconstruction

The images in the tilt series, tiltser_PtNP.tif, were aligned using a centre of mass (CM) alignment method after background subtraction and removal^15^. We present a reconstruction of this data, recon_PtNP.tif, produced using the equal slope tomography (EST) iterative algorithm, a method described by Miao *et al*.^23^ No Fourier filters were applied to the final reconstruction. Data illustrated in Fig. 2b.

### Tom_4: Atomic resolution tomography of tungsten needle

#### Sample preparation

A 99.95% pure tungsten wire was annealed under tension, creating a large crystalline domain with the [011] crystallographic axis aligned along the wire axis. The wire was electrochemically etched in a NaOH solution to form a sharp tip with a <10 nm diameter. The wire was plasma cleaned in an Ar/O_2_ gas mixture and then heated to 1,000 °C under vacuum (~10−5 Pa) to remove the oxide layer generated by the plasma cleaning. The wire was mounted in a 1 mm sample puck compatible with the TEAM microscope stage.

#### Data acquisition

Tomographic data was acquired using the TEAM I at the National Center for Electron Microscopy. The microscope was operated at 300 kV beam voltage in ADF-STEM mode with a convergence semi-angle of ~ 30 mrad and a ~ 70 pA beam current. The tomography rotation axis was aligned to the wire axis [011]. An equally sloped tomographic tilt series of 62 images, covering the complete angular range of ±90° was acquired from the tungsten needle sample. Two images of 1024×1024 pixels each with 6 μs per pixel dwell time and 0.405 Å pixel resolution were acquired at each angle in order to correct for drift. The TEAM stage, which is a tilt-rotate design with full 360° rotation about both axes, enabled rotation around the [011] crystalline axis.

#### Alignment and reconstruction

Raw experimental data can be found in tiltser_W.zip, which contains tif stacks of the two images acquired at each viewing angle, as described above. In addition, we provide an aligned tilt series, tiltser_W.tif. In the raw data, the tilt axis has a different in-plane orientation at each viewing angle, and in order to obtain an aligned tilt series, this was corrected by using Fourier methods to align the tilt direction along the image horizontal in every image. Both sample drift and scan distortion were corrected for in all images in the tilt series using Fourier techniques^34^. The tilt series was then aligned using a centre of mass method, with a mask applied to remove background noise. The tilt series was cropped in order to only feature the tip of the needle, which remained within the depth of focus throughout data acquisition. We present a reconstruction of the data, recon_W.tif, produced from tiltser_W.tif using the equal slope tomography iterative algorithm. The alignment and reconstruction process is explained in detail by Xu *et al*.^16^ Data illustrated in Fig. 2c.

### Tom_5: Through-focal tomography of Pt-Cu catalyst

#### Sample preparation

The through focal tilt series was acquired on PtCu nanoparticles on a 3D Vulcan carbon support. The synthesis methods and scientific relevance of the nanoparticles as a fuel-cell electrocatalyst are discussed in detail by Wang *et al.*^35^ To prepare for observation in the electron microscope, the particles were suspended in ethanol and pipetted onto a copper TEM grid with an ultra-thin, holey carbon support film.

#### Data acquisition

The through-focal tomographic tilt series of de-alloyed PtCu nanoparticles on an extended 3D carbon support was acquired using TEAM I at the National Center for Electron Microscopy; a tool that provides attributes to best demonstrate the advantages of this technique. Its large convergence angle provides high lateral resolution (<0.78 Å) and a small depth-of-field (~6 nm) at 300 kV accelerating voltage. Shadowing from the TEM grid limited tilts from −68° to +71° along our chosen axis of rotation.

**Figure 3:**
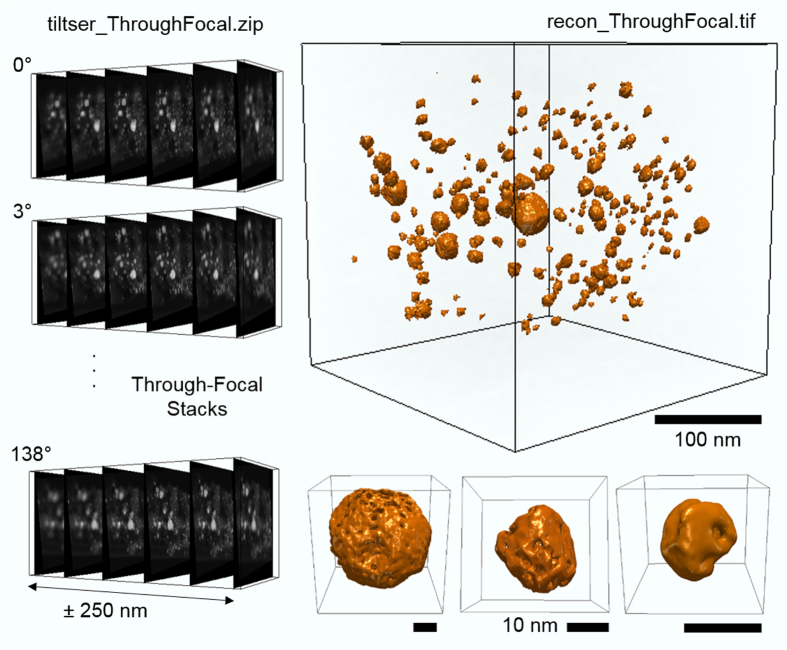
Illustration of raw data and sample reconstruction for Tom_5. A through-focal image series must be acquired at each viewing angle in through-focal tomography. Files 018.tif, 072.tif, and 120.tif are shown as examples. Through-focal tomography allows objects from an extended field of view to be reconstructed at high resolution in an aberration corrected STEM. A 3D isosurface visualization of the full view of PtCu nanoparticles on an extended carbon support in recon_ThroughFocal.tif is shown, along with high resolution 3D visualizations of individual PtCu particles in the reconstruction. All visualizations produced using tomviz.

The tomographic data was acquired over a 138° tilt range using a high angle annular dark field (HAADF) detector. The 30 mrad convergence angle provided a continuum of information in the through-focal CTF that spanned a ±1.72° wedge at low and medium frequencies. A 3° tilt increment was chosen to match the convergence angle. The PtCu nanoparticles decorate a 3D Vulcan carbon support with an extended structure that far exceeds the microscope’s depth of field—making it impossible to image multiple particles in-focus within a single field of view. At every tilt a 26 image through-focal series was taken over ±250 nm defocus with 20 nm focal steps in order to ensure all objects were imaged in focus. The microscope defocus steps are calibrated from a through-focal stack (Fig. 3). Each image had a 0.38 nm per pixel lateral resolution.

#### Alignment and reconstruction

A five-dimensional alignment of the raw data in tiltser_ThroughFocal.zip was required: transverse x-y alignment, focal z-alignment, tilt axis rotation and shift. A fiduciary particle was used to align each through-focal stack in their respective x-y direction. The focal z-alignment for each focal stack was determined by identification of the best focus image to a fiduciary particle. Within each focal stack a cross-correlation alignment was used to reduce the small amounts of drift during the acquisition. After alignment, the data was reweighted in Fourier space by dividing with the microscope’s contrast transfer function (CTF) approximated by a 300 keV 30 mrad aberration-free probe plus a Wiener constant of 5 times the max CTF value. After this light deconvolution, each through-focal stack was mapped onto a universal Fourier space by bilinear extrapolation. This extrapolation distributes the complex value of an input point to its four nearest neighbors on the output Cartesian grid with a weighted average of points from all the through-focal stacks. A direct inverse 3D Fourier transform provided the final reconstruction, recon_ThroughFocal.tif. This method is described by Hovden *et al.*^13^. It should be noted that the alignment of each through-focal stack generated excess blank images for reference. Thus in the raw data provided, there are 43 images per stack; 26 images of the PtCu nanoparticles, and 17 blank reference images.

### Code availability

Code equivalent to that used to reconstruct the data in Tom_1 and Tom_2 is available as part of the open source Tomviz software package at www.tomviz.org. Code used to reconstruct the data in Tom_3 is freely available online at http://www.physics.ucla.edu/research/imaging/EST/. Code used to reconstruct the data in Tom_4 is freely available online at http://www.physics.ucla.edu/research/imaging/3Datoms.

Code used to reconstruct the data in Tom_5 is available in the Supplementary Information to this paper (Supplementary File 1). Alignment tools are available as part of the open source Tomviz software package at www.tomviz.org, and the open source IMOD software package at http://bio3d.colorado.edu/imod/.

## Data Records

The datasets described in this paper are available at Figshare (Data Citation 1). All files are provided in 16-bit tif image format. Table 1 describes the content of the raw tilt series datasets. Table 2 describes tilt series derived from raw data, from which reconstructions are in turn derived, and Table 3 describes the sample reconstructions derived from the raw datasets that we have provided. All tilt series have their axis of rotation along the x-axis (horizontal) of the images.

### Further information on Tom_2: 180 degree tomography of NPs on nanofibre

tiltser_180.zip contains 190.tif stacks. Each stack is a series of sixteen images of one viewing angle, with each image acquired at 1 μs per pixel dwell time. The images in the each of stacks have been aligned by cross correlation. The image stacks have also been aligned with each other, allowing users to construct their own aligned tilt series from the image stacks. A full file listing for tiltser_180.zip is given below. Roman numerals indicate a stack from the first or the second tilt series. The number in the label indicates the viewing angle given by the microscope goniometer.


    I_00.tif    I_12.tif    I_-23.tif   I_35.tif    I_-46.tif
    I_01.tif    I_-12.tif   I_24.tif    I_-35.tif   I_47.tif
    I_-01.tif   I_13.tif    I_-24.tif   I_36.tif    II_00.tif
    I_02.tif    I_-13.tif   I_25.tif    I_-36.tif   II_01.tif
    I_-02.tif   I_14.tif    I_-25.tif   I_37.tif    II_-01.tif
    I_03.tif    I_-14.tif   I_26.tif    I_-37.tif   II_02.tif
    I_-03.tif   I_15.tif    I_-26.tif   I_38.tif    II_-02.tif
    I_04.tif    I_-15.tif   I_27.tif    I_-38.tif   II_03.tif
    I_-04.tif   I_16.tif    I_-27.tif   I_39.tif    II_-03.tif
    I_05.tif    I_-16.tif   I_28.tif    I_-39.tif   II_04.tif
    I_-05.tif   I_17.tif    I_-28.tif   I_40.tif    II_-04.tif
    I_06.tif    I_-17.tif   I_29.tif    I_-40.tif   II_05.tif
    I_-06.tif   I_18.tif    I_-29.tif   I_41.tif    II_-05.tif
    I_07.tif    I_-18.tif   I_30.tif    I_-41.tif   II_06.tif
    I_-07.tif   I_19.tif    I_-30.tif   I_42.tif    II_-06.tif
    I_08.tif    I_-19.tif   I_31.tif    I_-42.tif   II_07.tif
    I_-08.tif   I_20.tif    I_-31.tif   I_43.tif    II_-07.tif
    I_09.tif    I_-20.tif   I_32.tif    I_-43.tif   II_08.tif
    I_-09.tif   I_21.tif    I_-32.tif   I_44.tif    II_-08.tif  
    I_-10.tif   I_22.tif    I_-33.tif   I_45.tif    II_-09.tif
    I_11.tif    I_-22.tif   I_34.tif    I_-45.tif   II_10.tif
    I_-11.tif   I_23.tif    I_-34.tif   I_46.tif    II_-10.tif
    II_11.tif   II_-18.tif  II_26.tif   II_-33.tif  II_41.tif
    II_-11.tif  II_19.tif   II_-26.tif  II_34.tif   II_-41.tif
    II_12.tif   II_-19.tif  II_27.tif   II_-34.tif  II_42.tif
    II_-12.tif  II_20.tif   II_-27.tif  II_35.tif   II_-42.tif
    II_13.tif   II_-20.tif  II_28.tif   II_-35.tif  II_43.tif
    II_-13.tif  II_21.tif   II_-28.tif  II_36.tif   II_-43.tif
    II_14.tif   II_-21.tif  II_29.tif   II_-36.tif  II_44.tif
    II_-14.tif  II_22.tif   II_-29.tif  II_37.tif   II_-44.tif
    II_15.tif   II_-22.tif  II_30.tif   II_-37.tif  II_45.tif
    II_-15.tif  II_23.tif   II_-30.tif  II_38.tif   II_-45.tif
    II_16.tif   II_-23.tif  II_31.tif   II_-38.tif  II_46.tif
    II_-16.tif  II_24.tif   II_-31.tif  II_39.tif   II_-46.tif
    II_17.tif   II_-24.tif  II_32.tif   II_-39.tif  II_47.tif
    II_-17.tif  II_25.tif   II_-32.tif  II_40.tif   II_-47.tif
    II_18.tif   II_-25.tif  II_33.tif   II_-40.tif  II_48.tif


### Further information on Tom_3: Atomic resolution tomography of platinum nanoparticle

The images in tiltser_PtNP.tif were acquired using the equal slope tomography method. Rather than acquiring images at a fixed angular increments as in traditional tomography, the images are acquired at viewing angles that give equal increments of the slope of the Fourier transformed image planes in Fourier space^23^. The viewing angles (in degrees) associated with each of the 109 images in the tif stack are listed below:


    72.646  45.000  17.354  −20.556 −46.848
    71.030  44.091  15.709  −22.109 −47.816
    69.44   43.152  14.036  −23.629 −48.814
    67.891  42.184  12.339  −25.115 −49.844
    66.371  41.186  10.62   −26.565 −50.906
    64.885  40.156  8.8807  −27.979 −52.001
    63.435  39.094  7.125   −29.358 −53.130
    62.021  37.999  5.3558  −30.700 −54.293
    60.642  36.870  3.5763  −32.005 −55.491
    59.300  35.707  1.7899  −33.275 −56.725
    57.995  34.509  0.0000  −34.509 −57.995
    56.725  33.275  −1.7899 −35.707 −59.300
    55.491  32.005  −3.5763 −36.870 −60.642
    54.293  30.700  −5.3558 −37.999 −62.021
    53.130  29.358  −7.1250 −39.094 −63.435
    52.001  27.979  −8.8807 −40.156 −64.885
    50.906  26.565  −10.620 −41.186 −66.371
    49.844  25.115  −12.339 −42.184 −67.891
    48.814  23.629  −14.036 −43.152 −69.444
    47.816  22.109  −15.709 −44.091 −71.030
    46.848  20.556  −17.354 −45.000 −72.646
    45.909  18.970  −18.970 −45.909


### Further information on Tom_4: Atomic resolution tomography of tungsten needle

The images in tiltser_W.zip were acquired using the equal slope tomography method. Rather than acquiring images at a fixed angular increments as in traditional tomography, the images are acquired at viewing angles that give equal increments of the slope of the Fourier transformed image planes in Fourier space^23^. The viewing angles (in degrees) associated with each of the 63 image stacks is given in the filename of each image. A list of files in tiltser_W.zip is given below. In order to obtain a reconstruction from the raw images, they must be combined into a tilt series, and aligned (see Tom_4 Atomic Resolution Tomography of Tungsten Needle under Methods).


    0.tif       39.tif      69.3.tif    116.4.tif   147.8.tif
    3.5.tif     41.1.tif    72.5.tif    119.2.tif   150.5.tif
    7.1.tif     43.1.tif    75.9.tif    121.9.tif   153.3.tif
    10.6.tif    44.9.tif    79.3.tif    124.4.tif   156.2.tif
    14.tif      46.8.tif    82.7.tif    126.7.tif   159.1.tif
    17.3.tif    48.8.tif    86.3.tif    128.9.tif   159.3.tif
    20.5.tif    50.8.tif    89.8.tif    131.1.tif   164.3.tif
    23.6.tif    53.1.tif    93.5.tif    134.9.tif   167.8.tif
    26.5.tif    55.4.tif    97.tif      136.7.tif   168.tif
    29.3.tif    57.9.tif    100.4.tif   138.7.tif   175.9.tif
    32.tif      60.6.tif    103.9.tif   140.8.tif   180.tif
    34.5.tif    63.3.tif    107.2.tif   143.tif
    36.8.tif    66.3.tif    110.4.tif   145.3.tif


### Further information on Tom_5: Through-focal tomography of Pt-Cu catalyst

tiltser_ThroughFocal.zip contains 47 tif stacks. Each stack is an individual through focal series taken at a different viewing angle. Each of these tif stacks contains 43 images, 26 images of the sample, and 17 blank reference images for through-focal alignment. A reconstruction incorporating all of the information available in the data must be produced directly from the images in each of the 47 image stacks, rather than by combining the stacks into a single file tilt series as for the datasets above.

The title of each tif stack is the viewing angle in degrees that the through focal series was acquired at, measured from the first viewing angle. For example, the first through-focal tif stack, taken at 0°, is named 000.tif. The second tif stack, taken at a tilt of 3° relative to the first is labelled 003.tif. The focal increment between each image in each tif stack is 20 nm.

A full file listing for tiltser_ThroughFocal.zip is given below:


000.tif     030.tif     060.tif     090.tif     120.tif
003.tif     033.tif     063.tif     093.tif     123.tif
006.tif     036.tif     066.tif     096.tif     126.tif
009.tif     039.tif     069.tif     099.tif     129.tif
012.tif     042.tif     072.tif     102.tif     132.tif
015.tif     045.tif     075.tif     105.tif     135.tif
018.tif     048.tif     078.tif     108.tif     138.tif
021.tif     051.tif     081.tif     111.tif
024.tif     054.tif     084.tif     114.tif
027.tif     057.tif     087.tif     117.tif


Image 39 in stack 120.tif was not used as part of the direct Fourier reconstruction of the data because it was observed to contain a large scan distortion.

## Technical Validation

The electron microscopes used to acquire the datasets described in this paper were professionally maintained and aligned for optimal imaging conditions prior to dataset acquisition. For Tom_1, Tom_2, Tom_3, and Tom_4 an appropriately sized C2 aperture was selected for data acquisition in order to produce a depth of field that extended over the entire height of the object (or section of the object) to be imaged and reconstructed. For Tom_5 a small depth of field enhanced the through focal technique by providing more 3D information at every tilt. The images in each tilt series presented in this paper have been aligned. The high quality sample reconstructions we provide validate the accuracy and quality of the raw data. No obvious signs of morphological distortions that would indicate poor data acquisition are visible in either the raw data or in the reconstructions themselves. Sinograms of the reconstruction were also inspected for proper alignment to ensure that reconstructions of the highest quality were obtained^18^.

## Usage Notes

Collectively, these tomographic datasets of nanoscale materials provide a standard for the development and validation of new 3D imaging methods—from alignment, to reconstruction, to visualization and analysis. Their uses are diverse. The tilt series data can be intentionally degraded by adding misalignment or noise to explore its influence on a particular reconstruction algorithm. In Tom_2 true experimental noise can be added by discarding images within each tilt, thereby reducing the signal to noise ratio. The effects of increasing missing wedge size or tilt increment size can be explored by removing projections from the 180° tilt range in Tom_2 or Tom_4. The high resolution of data in Tom_3 and Tom_4 provide lattice peaks in the 3D Fourier transform of the final reconstruction. These lattice peaks may have appeal to understanding post processing filters. Lastly, Tom_5 provides exploration the limited depth of field that accompanies a new generation of aberration-electron microscopes and its influence on tomography. Each reconstruction provides a playground for visualization and standards for comparison. Tom_1 in particular has an intricate morphology and aesthetic beauty.

This manuscript illustrates the steps necessary to acquire, align, and reconstruct data from nanoscale specimens at the highest quality. The educational utility of the openly available datasets presented here toward training new scientists in electron tomography should not be understated.

The tilt-series data is best viewed using 2D image processing software such as ImageJ, Fiji, or Cornell Spectrum Imager^36^. The reconstructed datasets require 3D visualization software. The open source software tomviz (www.tomviz.org) was used to produce the data visualizations included in this paper^37^. Alternatives include the free to use UCSF Chimera and commercial tools. Datasets and reconstructions may be viewed on Windows, Mac OSX, and Linux operating systems. We recommend a RAM of at least twice the size of the file being viewed for best performance.

## Additional information

**How to cite this article:** Levin, B. D. A. *et al.* Nanomaterial datasets to advance tomography in scanning transmission electron microscopy. *Sci. Data* 3:160041 doi: 10.1038/sdata.2016.41 (2016).

## Supplementary Material

Supplementary File 1

## Figures and Tables

**Table 1:** Raw tilt series metadata.

**Data ID**	**Raw Dataset**	**Tomography Method**	**No. Images (Stacks)**	**Image Size (Pixels)**	**Angle Step**	**Angle Range**	**Pixel Size**
Tom_1	tiltser_Co2P.tif	Traditional	76 (1)	1157×1157	2°	146°	0.71 nm
Tom_2	tiltser_180.zip	180°	3040 (190)	947×1033	1°	I) 93° II) 108°	0.36 nm
Tom_3	tiltser_PtNP.tif	EST	109 (1)	401×401	N/A	145°	0.035 nm
Tom_4	tiltser_W.zip	EST	126 (63)	1024×1024	N/A	180°	0.0405 nm
Tom_5	tiltser_ThroughFocal.zip	Through-Focal	2021 (47)	1145×1145	3°	138°	0.38 nm

**Table 2:** Derived tilt series metadata.

**Data ID**	**Derived Dataset (Tilt Series)**	**Data Processing Methods**	**No. Images**	**Image Size (Pixels)**	**Pixel Size**
Tom_2	tiltser_180.tif	Sum and combine image stacks.	180	947×1033	0.36 nm
Tom_4	tiltser_W.tif	Align images. Correct drift and distortions. Sum and combine image stacks. Remove noise. Crop tilt series.	62	54×331	0.0405 nm
Tilt increments (angle step) and angular ranges as in Table 1 above.					

**Table 3:** Derived reconstruction metadata.

**Data ID**	**Derived Dataset (Reconstruction)**	**Reconstruction Method**	**Reconstruction Size (Voxels)**	**Voxel Size**
Tom_1	recon_Co2P.tif	SIRT	579×579×579	1.42 nm
Tom_2	recon_180.tif	WBP	517×517×522	0.72 nm
Tom_3	recon_PtNP.tif	EST Iterative Algorithm	241×241×241	0.058 nm
Tom_4	recon_W.tif	EST Iterative Algorithm	255×255×105	0.053 nm
Tom_5	recon_ThroughFocal.tif	Direct Fourier Transform	1025×1025×1025	0.38 nm
