# Supplementary material for: Nanomaterial datasets to advance tomography in scanning transmission electron microscopy
Source: Sci Data. 2016 Jun 7;3:160041. doi: 10.1038/sdata.2016.41 (PMC4896123; doi:10.1038/sdata.2016.41)
Supplement: Supplementary File 1 [file sdata201641-s1.doc]

SUPPLEMENTARY INFORMATION for

Nanomaterial datasets to advance tomography in scanning transmission electron microscopy

Barnaby D.A. Levin^1^, Elliot Padgett^1^, Chien-Chun Chen^2,3^, Mary C. Scott^2,4^, Rui Xu^2^, Wolfgang Theis^5^, Yi Jiang^6^, Yongsoo Yang^2^, Colin Ophus^4^, Haitao Zhang^7^, Don-Hyung Ha^7^, Deli Wang^8,9^, YingchaoYu^8^, Hector D. Abruña^8^, Richard D. Robinson^7^, Peter Ercius^4^, Lena F. Kourkoutis^1,10^, Jianwei Miao^2^ , David A. Muller^1,10^, Robert Hovden^1^.

1. School of Applied and Engineering Physics, Cornell University, Ithaca, New York, 14853, USA.
2. Department of Physics & Astronomy, and California NanoSystems Institute University of California, Los Angeles, California, 90095, USA.
3. Department of Physics, National Sun Yat-Sen University, Kaohsiung, 80424, Taiwan.
4. National Center for Electron Microscopy, Molecular Foundry, Lawrence Berkeley National Laboratory, Berkeley, California, 94720, USA.
5. Nanoscale Physics Research Laboratory, School of Physics and Astronomy, University of Birmingham, Edgbaston, Birmingham, B15 2TT, UK.
6. Department of Physics, Cornell University, Ithaca, New York, 14853, USA.
7. Department of Materials Science and Engineering, Cornell University, Ithaca, New York, 14853, USA.
8. Department of Chemistry and Chemical Biology, Cornell University, Ithaca, New York, 14853, USA
9. School of Chemistry and Chemical Engineering, Huazhong University of Science and Technology, Wuhan, 430074, China.
10. Kavli Institute for Nanoscale Science, Cornell University, Ithaca, New York, 14853, USA.

**Code to reconstruct a through-focal tomography dataset**

The following MATLAB script will reconstruct the raw data in dataset Tom_5.

%through_focal_recon.m

%MATLAB script to perform through focal reconstruction on tilt series in Tom_5.

%File has to be in the same folder as all the data.

%Final image is stored as variable "recon", which can then be written.

%%%%%%%%%%%%%%%%%%%%%%%%%%%%%%%%%%%%%%%%%%%%%%%%%%%%%%%%%%%%%%%%%%%%%%%%%%%

%Parameters

%Size of the output volume

N = 255; %smaller field of view for faster computation

cen_r = floor(N/2)+1; %index of origin

r = cen_r-1;

%Location of the fiducial particle

lx = 570;

ly = 780;

%number of images in through focal direction

N_z = 127;

cen_z = floor(N_z/2)+1;

%Transfer function

dr = 436.69/1145*10; %angstrom

dz = 150; %angstrom

delta_k_r=1/(N*dr);

delta_k_z=1/(N_z*dz);

%Probe function

alpha_max = 30e-3; %semi-convergence angle in rad

Voltage = 300; %keV

lambda = 12.3986./sqrt((2*511.0+Voltage).*Voltage); %wavelength in angstrom

%%%%%%%%%%%%%%%%%%%%%%%%%%%%%%%%%%%%%%%%%%%%%%%%%%%%%%%%%%%%%%%%%%%%%%%%%%%

fprintf('Initializingn...');

wiener_filter = 0;

%Set up Fourier space coord.

kx = single(linspace(-floor(N/2),ceil(N/2)-1,N));

ky = single(linspace(-floor(N/2),ceil(N/2)-1,N));

kz = single(linspace(-floor(N_z/2),ceil(N_z/2)-1,N_z));

[kX,kY,kZ]= meshgrid(kx,ky,kz);

kX = kX *delta_k_r;

kY = kY *delta_k_r;

kZ = kZ *delta_k_z;

%First, make a mask function (a bit smaller than the actual CTF)

H = 2./(pi*alpha_max*0.9*sqrt(kX.^2+kY.^2)).* sqrt(1-( abs(kZ)./(sqrt(kX.^2+kY.^2)*alpha_max*0.9) + sqrt(kX.^2+kY.^2)*lambda/(2*alpha_max*0.9)).^2);

mask = imag(H)==0;

mask(cen_r,cen_r,cen_z)=1;

H = 2./(pi*alpha_max*sqrt(kX.^2+kY.^2)).* sqrt(1-( abs(kZ)./(sqrt(kX.^2+kY.^2)*alpha_max) + sqrt(kX.^2+kY.^2)*lambda/(2*alpha_max)).^2);

H(cen_r,cen_r,:)=0;

H = single(H.*single(imag(H)==0 ));

H(cen_r,cen_r,cen_z)=max(max(max(H)));

H_max = max(max(max(H)));

H(H~=0) = (H(H~=0).^2+H_max^2*wiener_filter)./H(H~=0);

H = H.*single(mask);

fprintf('Done!\n');

%set up Fourier space coord.

kx = linspace(cen_r-N,N-cen_r,N);

ky = linspace(cen_r-N,N-cen_r,N);

kz = linspace(cen_z-N_z,N_z-cen_z,N_z );

kz = kz/(dz/dr);

[kX, kY, kZ] = meshgrid(kx,ky,kz);

%matrices for bilinear extrapolation

w = ones(N,N,N)*eps;

v = zeros(N,N,N);

%%%%%%%%%%%%%%%%%%%%%%%%%%%%%%%%%%%%%%%%%%%%%%%%%%%%%%%%%%%%%%%%%%%%%%%%%%%

%Reconstruction

for a = 1:47

%Import stack

fprintf(strcat('Importing stack No.',int2str(a),'...'));

%Calculate angle

ang = (a-1)*3; %degree

if ang<10

name = strcat('00',int2str(ang),'.tif');

else

if ang<100

name = strcat('0',int2str(ang),'.tif');

else

name = strcat(int2str(ang),'.tif');

end

end

info = imfinfo(name);

N_s = numel(info); % number of images in each stack

%Read tiff file

%Read the first image to determine the size of the image

data = imread(name, 1, 'Info', info);

%Allocate memory of stack by padding zeros

data = padarray(data,[0 0 N_s-1], 'post');

%Read the rest of images in the stack

for i = 2:N_s

data(:,:,i) = imread(name, i, 'Info', info);

end

data = double(data);

%Delete the bad imagae

if a==31

data(:,:,37:end) = 0;

end

fprintf('Done!\n');

fprintf('Processing data...');

%Change the size in z direction

if N_z >N_s

data = padarray(data,[0 0 (N_z-1)/2 - (N_s-1)/2]);

else

data = data(:,:,(N_s-N_z)/2+1:(N_s+N_z)/2);

end

%Change the size in xy direction

N_xy = size(data,1);

temp = zeros(N,N,N_z);

if N_xy>=N

for i=1:N_z

temp(:,:,i) = data(lx-r:lx+r,ly-r:ly+r,i); %crop data

end

else

temp = padarray(data,[(N-N_xy)/2 (N-N_xy)/2 0]); %pad zeros to image

end

data = temp;

fprintf('Mapping data to Fourier space...');

%FFT data

F = fftshift(fftn(ifftshift(data)));

F = F.*mask;

F(F~=0) = F(F~=0)./conj(H(F~=0));

ang = -((a-1)*3-68)*pi/180;

kY_new = cos(ang)*kY - sin(ang)*kZ;

kZ_new = sin(ang)*kY + cos(ang)*kZ;

sY = abs(floor(kY_new) - kY_new);

sZ = abs(floor(kZ_new) - kZ_new);

for i = 1:N

for j = 1:N

for k = 1:N_z

value = F(i,j,k);

if value~=0

%P1

p_x = kX(i,j,k)+cen_r;

p_y = floor(kY_new(i,j,k))+cen_r;

p_z = floor(kZ_new(i,j,k))+cen_r;

if p_x >0 && p_x<=N && p_y >0 && p_y<=N && p_z >0 && p_z<=N

w(p_y,p_x,p_z) = w(p_y,p_x,p_z) + (1-sY(i,j,k))*(1-sZ(i,j,k));

v(p_y,p_x,p_z) = v(p_y,p_x,p_z) + (1-sY(i,j,k))*(1-sZ(i,j,k)) * value;

end

%P2

p_x = kX(i,j,k)+cen_r;

p_y = ceil(kY_new(i,j,k))+cen_r;

p_z = floor(kZ_new(i,j,k))+cen_r;

if p_x >0 && p_x<=N && p_y >0 && p_y<=N && p_z >0 && p_z<=N

w(p_y,p_x,p_z) = w(p_y,p_x,p_z) + sY(i,j,k)*(1-sZ(i,j,k));

v(p_y,p_x,p_z) = v(p_y,p_x,p_z) + sY(i,j,k)*(1-sZ(i,j,k)) * value;

end

%P3

p_x = kX(i,j,k)+cen_r;

p_y = floor(kY_new(i,j,k))+cen_r;

p_z = ceil(kZ_new(i,j,k))+cen_r;

if p_x >0 && p_x<=N && p_y >0 && p_y<=N && p_z >0 && p_z<=N

w(p_y,p_x,p_z) = w(p_y,p_x,p_z) + (1-sY(i,j,k))*sZ(i,j,k);

v(p_y,p_x,p_z) = v(p_y,p_x,p_z) + (1-sY(i,j,k))*sZ(i,j,k)* value;

end

%P4

p_x = kX(i,j,k)+cen_r;

p_y = ceil(kY_new(i,j,k))+cen_r;

p_z = ceil(kZ_new(i,j,k))+cen_r;

if p_x >0 && p_x<=N && p_y >0 && p_y<=N && p_z >0 && p_z<=N

w(p_y,p_x,p_z) = w(p_y,p_x,p_z) +sY(i,j,k)*sZ(i,j,k);

v(p_y,p_x,p_z) = v(p_y,p_x,p_z) +sY(i,j,k)*sZ(i,j,k) * value;

end

end

end

end

end

fprintf('Done!\n');

end

%Taking inverse FFT

temp1= v./w;

temp2 = flipdim(flipdim(flipdim(temp1,1),2),3);

recon = ((real(temp1)+real(temp2))/2 + (imag(temp1)-imag(temp2))/2 .* 1i );

recon = fftshift(ifftn(ifftshift(recon)));
